# Approaching a network connectivity-driven classification of the psychosis continuum: a selective review and suggestions for future research

**DOI:** 10.3389/fnhum.2014.01047

**Published:** 2015-01-13

**Authors:** André Schmidt, Vaibhav A. Diwadkar, Renata Smieskova, Fabienne Harrisberger, Undine E. Lang, Philip McGuire, Paolo Fusar-Poli, Stefan Borgwardt

**Affiliations:** ^1^Department of Psychiatry (UPK), University of BaselBasel, Switzerland; ^2^Department of Psychiatry and Behavioral Neurosciences, Wayne State UniversityDetroit, Michigan, USA; ^3^Department of Psychosis Studies, Institute of Psychiatry, King's College LondonLondon, UK

**Keywords:** psychosis continuum, fronto-parietal connectivity, structural connectivity, functional connectivity, fMRI, DTI, dynamic causal modeling, graph theory

## Abstract

Brain changes in schizophrenia evolve along a dynamic trajectory, emerging before disease onset and proceeding with ongoing illness. Recent investigations have focused attention on functional brain interactions, with experimental imaging studies supporting the disconnection hypothesis of schizophrenia. These studies have revealed a broad spectrum of abnormalities in brain connectivity in patients, particularly for connections integrating the frontal cortex. A critical point is that brain connectivity abnormalities, including altered resting state connectivity within the fronto-parietal (FP) network, are already observed in non-help-seeking individuals with psychotic-like experiences. If we consider psychosis as a continuum, with individuals with psychotic-like experiences at the lower and psychotic patients at the upper ends, individuals with psychotic-like experiences represent a key population for investigating the validity of putative biomarkers underlying the onset of psychosis. This paper selectively addresses the role played by FP connectivity in the psychosis continuum, which includes patients with chronic psychosis, early psychosis, clinical high risk, genetic high risk, as well as the general population with psychotic experiences. We first discuss structural connectivity changes among the FP pathway in each domain in the psychosis continuum. This may provide a basis for us to gain an understanding of the subsequent changes in functional FP connectivity. We further indicate that abnormal FP connectivity may arise from glutamatergic disturbances of this pathway, in particular from abnormal NMDA receptor-mediated plasticity. In the second part of this paper we propose some concepts for further research on the use of network connectivity in the classification of the psychosis continuum. These concepts are consistent with recent efforts to enhance the role of data in driving the diagnosis of psychiatric spectrum diseases.

## Introduction

Almost two decades ago, it was proposed that the pathophysiology of psychosis is more closely related to abnormal functional integration between specific brain regions rather than to changes in local brain activity (Friston and Frith, 1995; Friston, 1998). Numerous experimental investigations using functional magnetic resonance imaging (fMRI) and diffusion tensor imaging (DTI) have supported the disconnection hypothesis by showing abnormal structural and functional connectivity between a wide range of brain regions and across different stages of the psychosis spectrum (Kyriakopoulos and Frangou, 2009; Peters et al., 2010; Pettersson-Yeo et al., 2011; Yao et al., 2013; Samartzis et al., 2014). These findings provide evidence that abnormal brain connectivity develops even before the onset of psychosis and evolves along the psychosis continuum, which suggests that the assessment of the brain connectivity pattern may permit the detection of the early phases of the illness (Schmidt and Borgwardt, 2013). One of the most consistent findings is a reduction in FP connectivity (whether structural or functional) across all stages in the psychosis spectrum (Pettersson-Yeo et al., 2011), and this suggests that abnormalities in this pathway reflect a vulnerability marker for emerging psychosis (Schmidt et al., 2013, 2014a). Functional and structural interactions between FP brain regions are crucial for successful working memory (WM) processing (Gazzaley et al., 2004; Cole and Schneider, 2007; Gazzaley and Nobre, 2012) and it is therefore conceivable that abnormal FP connectivity underlies WM impairment in psychosis. These deficits in WM processing are of particular interest, as they precede the onset of psychosis by many years and in the absence of any psychotic symptom. They may therefore offer valuable predictions about the longitudinal course of the disease (Fusar-Poli et al., 2012a).

In the present article, we selectively review neuroimaging findings of FP connectivity across different stages of the psychosis continuum, including chronic psychosis, early psychosis, clinical high-risk patients, genetic high-risk patients, as well as the general population with psychotic symptoms (see Table 1 for the different phenotypes across the psychosis continuum). We used the term “psychosis” throughout the paper to refer to patients with schizophrenia spectrum psychoses, i.e., schizophrenia, schizophreniform disorder, schizoaffective, as well as other psychoses including unipolar or bipolar depression with psychotic symptoms. We first report structural FP connectivity findings (by means of fractional anisotropy values derived from DTI), as this may help the reader to better understand the findings on abnormal functional connectivity during the resting state and in WM-induced paradigms, which are presented subsequently. In the next brief section, we then provide evidence that abnormal FP connectivity may result from glutamatergic disturbances, in particular from changes in N-methyl-D-aspartate receptor (NMDAR)–dependent synaptic plasticity.

**Table 1:** **Different phenotypes across the psychosis continuum**.

**Phenotype**	**Conceptualization**
Patients with chronic psychotic disorder	Patients with clinical supra-threshold symptoms (assessed with DSM-5/ICD-10)
First episode of psychosis patients	Patients already fulfil criteria for acute psychotic disorder according to ICD-10 or DSM-IV but not yet for schizophrenia (Yung et al., 1998) First time patient experiences psychotic symptoms or a psychotic episode Can remit entirely after one episode or incompletely with persisting symptoms, or continue to chronic schizophrenia
Clinical high risk subjects - ultra high risk, at-risk mental state ^**^	Help-seeking people with clinical attenuated or brief limited psychotic symptoms Moderate but sub-threshold psychotic symptoms Moderate neurocognitive changes. Higher clinical risk to develop psychosis
Genetic high risk subjects	First or second degree relatives of psychotic patients Mostly non-clinical psychotic symptoms. Increased risk for psychosis or severe mood disorders
•Non-help-seeking individual from the general population with psychotic-like experiences^*^	Non-help-seeking subjects from the general population (ca. 8%) Occasional psychosis-like experiences Non-clinical symptoms Psychosis risk modest (Fusar-Poli et al., 2014c)

The risk for subsequent transition to psychosis increases from healthy subjects with occasional pre-psychotic signs to genetic and clinical high risk subjects.

* Often assessed with the CAPE (Community Assessment of Psychic Experiences) questionnaire (Stefanis et al., 2002);

** *Clinical definition criteria vary across centers (see Smieskova et al., 2010). DSM, Diagnostic and Statistical Manual of Mental Disorders, ICD, International Classification of Diseases*.

There are two main concepts as to how brain connectivity can be measured and modeled: one is based on graph theory describing the brain's network topology in terms of undirected connections (correlations) derived from structural or functional MRI. Graph theory analysis has emerged as a very helpful approach to infer complex network properties of the brain from a more global perspective. In this framework, brain networks can be understood as graphs that are composed of nodes representing neural elements, such as neurons or brain regions, that are linked by edges denoting structural (anatomical links) or functional connections. Their topological properties or patterns of connectivity can be expressed by a variety of parameters, such as node degree, clustering coefficient, path length, connection density, or modularity (Bullmore and Sporns, 2009; Rubinov and Sporns, 2010). For a comprehensive review of the contribution of modern network theory to the understanding of neurological diseases see Stam (2014). One of the major contributions of graph theory to our understanding of neurological and neuropsychiatric diseases has been in highlighting the important role of hubs, which are nodes of the network with an unusually high degree (number of connections). For details, see Rubinov and Bullmore (2013b), Van Den Heuvel and Sporns (2013), Crossley et al. (2014). These special nodes are often highly connected to each other, thus giving rise to the so-called rich-club organization. The second approach constitutes model-based formulations of context-specific effective (directed) connectivity that are based on the biophysics of neuronal interactions, such as dynamic causal modeling (DCM) (Park and Friston, 2013). In particular, DCM is a Bayesian identification scheme based on a model of neuronal interactions and an observation model (for fMRI, the haemodynamic model is used) (Friston et al., 2003). This is particularly useful in testing neurobiologically informed *a priori* hypotheses (different DCMs), which can be compared with model comparison procedures. Both of these approaches,—model-based investigations of effective connectivity and more global data-driven correlation approaches across the whole brain with graph theory—have already demonstrated network connectivity abnormalities in psychosis (Fornito et al., 2012; Dauvermann et al., 2014; Van Den Heuvel and Fornito, 2014) and are thus promising approaches to detect robust network connectivity endophenotypes for different stages of the psychosis continuum, although a number of challenges remain. In order to address these current limitations and challenges, in the last part of this paper we suggest some potential approaches in developing a network connectivity-driven classification of the psychosis continuum on the basis of non-invasive neuroimaging data.

## Structural changes in the FP pathway

### Chronic psychosis

Patients with chronic psychosis show significant intra-regional reductions in gray matter in FP brain regions (Marsh et al., 2001; Olabi et al., 2011; Kumra et al., 2012; Asami et al., 2013). The white matter bundle connecting FP brain regions, the superior longitudinal fasciculus, is also affected in chronic psychotic patients, which indicates reduced FP structural connectivity. Patients with psychosis manifest reduced fractional anisotropy values in the superior longitudinal fasciculus (Buchsbaum et al., 2006; Shergill et al., 2007; Karlsgodt et al., 2008; Kyriakopoulos et al., 2008; Seal et al., 2008; Clark et al., 2011; White et al., 2011; Filippi et al., 2014). In both patient and control groups, fractional anisotropy values in the superior longitudinal fasciculus were correlated with performance in a verbal working memory task (Karlsgodt et al., 2008), as well as with PANSS scores (Michael et al., 2008) and with the severity score of auditory hallucinations in schizophrenia patients (Seok et al., 2007). Changes in white matter, including the superior longitudinal fasciculus (Lebel et al., 2008), temporally coincide with cognitive development during adolescence and early adulthood, crucial periods in brain remodeling (Ashtari et al., 2007). Besides conventional analyses of structural brain abnormalities, graph theory analysis has emerged as a very helpful approach for inferring complex network properties of the brain and will play an increasingly important part in the effort to comprehend the physics of the brain's connectome (Bullmore and Sporns, 2009) and how this is affected in psychosis (Van Den Heuvel and Fornito, 2014). Using graph analysis, aberrant structural network properties within the frontal and temporal cortex have been found in psychosis patients, which suggests that psychosis impacts the global network connectivity of fronto-temporal brain regions (Van Den Heuvel et al., 2010). Disrupted axonal fiber connectivity in schizophrenia has been observed within a distributed network of nodes comprising frontal, parietal and temporal regions (Zalesky et al., 2011). By describing whole brain changes in structural connectivity, this study supports the macro-circuit theory of psychosis, which posits that specific white matter tracts are disrupted in psychosis, either as a cause or a consequence of a disorder in the gray matter regions they connect (Konrad and Winterer, 2008; Ellison-Wright and Bullmore, 2009). Another study indicated that the rich-club organization is also significantly affected in psychosis patients, accompanied by reduced density of rich-club connections, predominantly in the white matter pathways that link the midline frontal, parietal, and insular hub regions. The so-called rich-club phenomenon in networks is said to be present when the hubs of a network tend to be more densely connected among themselves than with nodes of a lower degree (Colizza et al., 2006). This term is in analogy with social systems, where highly connected people (who are “rich” in their social connections) form a highly interconnected club (Zhou and Mondragon, 2004). The reduction in rich-club density was found to be associated with lower levels of global communication capacity (Van Den Heuvel et al., 2013). This study provides new biological evidence that psychosis is characterized by selective disruption of structural brain connectivity among central hub regions of the brain, potentially leading to reduced communication capacity and altered functional brain dynamics (Van Den Heuvel et al., 2013).

### Early psychosis

Longitudinal studies show a progressive loss of frontal and parietal gray matter in first episode psychosis patients (Kasparek et al., 2009; Arango et al., 2012; Vita et al., 2012). Fractional anisotropy values in the superior longitudinal fasciculus are also reduced in patients with first episode psychosis (Federspiel et al., 2006; Witthaus et al., 2008; Kyriakopoulos et al., 2009; Luck et al., 2010; Pérez-Iglesias et al., 2010a,b; Guo et al., 2012). Notably, increased fractional anisotropy values in the superior longitudinal fasciculus of patients with chronic and first-episode psychosis have also been reported (Hoptman et al., 2008; Bora et al., 2011), whereas others either found white matter changes besides the superior longitudinal fasciculus across all stages of psychosis or no changes at all (Ellison-Wright and Bullmore, 2009; Kyriakopoulos and Frangou, 2009; Peters et al., 2010; Lu et al., 2011; White et al., 2011; Yao et al., 2013; Canu et al., 2014; Samartzis et al., 2014). Using graph theory, significantly decreased anatomical connectivity among FP brain regions has been observed in medication-naïve first episode patients compared to the controls (Zhang et al., 2014).

### Clinical high-risk samples

Reduced FP gray matter volumes have also been found in clinical high risk subjects (Borgwardt et al., 2007; Smieskova et al., 2010; Mechelli et al., 2011; Fusar-Poli et al., 2011a, 2012c). Moreover, longitudinal studies reported reductions in gray matter in FP regions in high risk patients with transition to psychosis, compared to those without transition (Pantelis et al., 2003; Borgwardt et al., 2008, 2011). Furthermore, several studies in clinical high risk subjects found reduced fractional anisotropy values in the superior longitudinal fasciculus (Karlsgodt et al., 2009; Peters et al., 2009; Bloemen et al., 2010; Carletti et al., 2012), which were even more reduced in clinical high risk subjects with a subsequent transition to psychosis, compared to those without transition (Bloemen et al., 2010). These findings suggest that structural FP dysconnectivity may already be present in early stages of the disease. The degree of aberrant FP connectivity may depend on the developmental stage of the subjects, the duration of illness and exposure to antipsychotic medication (Canu et al., 2014).

### Genetic highrisk samples

Genetic high risk individuals exhibit reduced gray matter volumes in FP brain regions (Smieskova et al., 2012b; Thermenos et al., 2013; Fusar-Poli et al., 2014b). The genetic high risk state is further associated with reduced fractional anisotropy values in the superior longitudinal fasciculus (Hoptman et al., 2008; Clark et al., 2011; Knöchel et al., 2012), although increased values have been reported as well (Hoptman et al., 2008). A very recent diffusion tensor imaging study showed that abnormal rich-club organization is already evident in unaffected siblings of schizophrenia patients if compared with healthy subjects, but less affected than in schizophrenia patients, which suggests that impaired rich-club connectivity is related to familial vulnerability for schizophrenia and may therefore reflect genetic vulnerability (Collin et al., 2013).

### Non-help-seeking individuals with psychotic-like experiences

There is currently little evidence on structural brain abnormalities in subjects with psychotic-like experiences. One study has found reduced gray matter volume in the parietal cortex (Modinos et al., 2010a). No study has yet explored structural connectivity among FP pathways in these subjects.

## FP resting state connectivity in the psychosis spectrum

### Chronic psychosis

Numerous functional magnetic resonance imaging (fMRI) studies of the resting state have shown reduced FP connectivity in chronic psychotic patients (Bluhm et al., 2007; Woodward et al., 2011; Zalesky et al., 2012; Baker et al., 2014; Ma et al., 2014). Reduced FP resting state connectivity in psychosis patients is correlated with disorganization symptoms (Rotarska-Jagiela et al., 2010), whereas the variability of prefrontal cortex dysconnectivity at rest predicts the severity of cognitive deficits (Cole et al., 2011). Furthermore, decreased regional FP activity during resting state has also been reported in psychosis (Liu et al., 2006), and might be partly interlinked with reductions in inter-regional FP connectivity. Indeed, such a correlation between intra-regional homogeneity and inter-regional connectivity has been demonstrated in psychotic patients using graph theoretical approaches (Zalesky et al., 2012).

Resting state graph network analysis in psychosis had further indicated decreased edge connectivity strengths in sub-networks of FP regions (Liu et al., 2008; Fornito et al., 2012; Zalesky et al., 2012). Psychosis is also accompanied by reduced degree and clustering in frontal and parietal cortical nodes of functional networks during rest (Lynall et al., 2010). A recent resting state fMRI network study in chronic patients identified a non-linear measure of functional connectivity, akin to mutual information. Particularly within the FP network, this provides greater discriminative power in the diagnosis of schizophrenia than the traditional correlation coefficient, suggesting that the combination of both linear and non-linear FP connectivity measures should be taken into account in research on schizophrenia and other psychiatric disorders (Su et al., 2013). Graph theoretical methods also permit exploration of the central role for flexible FP hubs in cognitive control, with interesting implications for psychosis (Cole et al., 2013). Such sophisticated analyses of whole-brain network connectivity promise to shed new light on how the frontal cognitive control system is affected in early phases of psychosis.

### Early psychosis

Resting-state fMRI studies in first episode psychosis gave ambiguous results on FP connectivity. While some evidence points toward decreased FP connectivity (Zhou et al., 2007), increased FP connectivity in first episode patients has also been reported (Lui et al., 2010). It is striking that patients who received risperidone treatment showed significantly decreased functional connectivity in the ventromedial prefrontal cortex to the parietal lobule, after treatment, and relative to pretreatment values and controls (Lui et al., 2010). For a review on resting state brain connectivity in chronic and first episode psychosis in general also see Yu et al. (2012).

### Clinical high risk samples

Studies on resting state functional connectivity in high risk populations is ongoing and evidence is therefore limited in the literature, in particular with respect to studies focusing on FP connectivity. Several investigations have been conducted to examine how the FP system interacts with other cognition-related networks. Interactions between brain systems such as the default mode and the FP cognitive system are critical for the flexibility of normal cognitive control and its disruption in pathological conditions (Cocchi et al., 2013). For instance, a recent study reported hyper-connectivity relative to healthy controls in the default network between a parietal seed region and prefrontal areas in clinical high risk subjects. This hyper-connectivity in the default network was associated with reduced connectivity in the task-related network comprising FP brain regions (Shim et al., 2010), and may indicate deficient FP capacity for appropriate cognitive processing. Indeed, activity in the default mode is inversely correlated with cognitive control (Lawrence et al., 2003; McKiernan et al., 2003). Such a reduced negative correlation between the default mode network and the task-positive network has been observed in other studies with clinical high risk patients (Fryer et al., 2013; Wotruba et al., 2013).

### Genetic high risk samples

In accordance with studies in clinical high risk subjects, the inverse correlation between the default mode network and the task-positive network is also diminished in genetic high risk individuals (Whitfield-Gabrieli et al., 2009). Thus, a resting state fMRI study found reduced inter-network connectivity between the FP network and the cingulo-opercular network and a cerebellar network in individuals with psychosis and their unaffected siblings and that these reductions were associated with both cognitive impairments and clinical symptoms (Repovs et al., 2011). This is important evidence, as it suggests that antagonism between the default mode and FP activity is mediated by the cingulo-opercular system (Bressler and Menon, 2010).

### Non-help-seeking individuals with psychotic-like experiences

In contrast to these reduced within- and between-network connectivity of the FP system in patients with chronic and first-episode psychosis and individuals at high-risk for psychosis, non-help-seeking individuals with psychotic-like experiences exhibit increased FP connectivity at rest (Orr et al., 2014). Increased resting state activity in temporal and frontal regions, as well as in the cingulum and parahippocampus, has also been detected in non-psychotic individuals with auditory verbal hallucinations (Diederen et al., 2013; Van Lutterveld et al., 2014). The increased FP connectivity in non-clinical subjects has been interpreted as a protective factor or resilience for psychosis (Orr et al., 2014). We will comment on this interpretation later in the article, after embedding the result in the context of other connectivity findings.

## FP connectivity during WM processing in the psychosis continuum

### Chronic psychosis

WM deficits are of great significance for the pathophysiology of psychosis, as they occur across the whole psychosis spectrum (Fusar-Poli et al., 2012a). For instance, WM impairment might effectively reflect expression of liability to psychosis, as adolescents at clinical high risk for psychosis performed significantly worse than control subjects on spatial WM (Smith et al., 2006). Moreover, recent meta-analyses suggested that it is possible to differentiate clinical high-risk individuals with a later transition to psychosis with respect to their behavioral WM performance (De Herdt et al., 2013). Successful WM processing involves functional integration among FP brain areas (Owen et al., 2005; Sauseng et al., 2005; Dosenbach et al., 2008), and the superior longitudinal fasciculus critically contributes to WM performance (Karlsgodt et al., 2010). There is convincing evidence that impaired WM processing in psychosis results from abnormal FP connectivity (Pettersson-Yeo et al., 2011; Dauvermann et al., 2014). In particular, reduced WM-related FP connectivity in psychosis has been shown using fMRI (Perlstein et al., 2001; Schlösser et al., 2003; Walter et al., 2007; Henseler et al., 2009, 2010; Karch et al., 2009; Meda et al., 2009), positron emission tomography (Kim et al., 2003), and electroencephalography (Peled et al., 2001), although some studies also reported increased FP connectivity (Callicott et al., 2000; Whitfield-Gabrieli et al., 2009). Moreover, using dynamic causal modeling, a model-based technique for the analysis of effective connectivity (Friston et al., 2003), and reduced WM-induced modulation of FP connectivity has been observed in psychotic patients (Deserno et al., 2012).

### Early psychosis

Studies in first episode psychosis patients revealed reduced FP activity during WM processing, (Schneider et al., 2007; Broome et al., 2009; Fusar-Poli et al., 2010; Smieskova et al., 2012a). Graph analysis also revealed task-specific connectivity impairment in FP systems related to cognitive control in patients with first episode psychosis (Fornito et al., 2011). In accordance with these findings, dynamic causal modeling studies showed reduced effective connectivity between frontal and parietal brain regions in first episode patients (Roiser et al., 2013; Schmidt et al., 2013). It is striking that the abnormal modulation of connectivity in first episode psychosis patients was normalized by treatment with antipsychotics (Schmidt et al., 2013).

### Clinical high risk samples

Clinical high risk subjects also show reduced WM-induced FP activation compared with healthy controls (Smieskova et al., 2012a), but the extent of reduction is less severe than in those with first-episode psychosis (Broome et al., 2009). It is intriguing that reduced WM-related prefrontal activation in clinical high-risk individuals is associated with a reduction in gray matter volume in the same area (Fusar-Poli et al., 2011b). Furthermore, in accordance with studies in chronic and first-episode patients, the WM-induced modulation of FP connectivity is also reduced in clinical high risk subjects (Schmidt et al., 2014b), whose connectivity strengths were intermediate between those of healthy controls and of first episode patients (Schmidt et al., 2013). These studies suggest that vulnerability to psychosis is associated with a progressive failure in the functional integration of frontal and parietal regions involved in WM processes.

### Genetic high risk samples

In contrast to these reduction in FP connectivity, genetic high risk subjects showed increased activation in FP regions during the WM encoding phase relative to the healthy control group (Choi et al., 2012). This result corresponds with previous evidence showing increased FP connectivity in genetic high risk subjects (Whalley et al., 2004; Delawalla et al., 2008), which has been interpreted as a compensatory mechanism (Whalley et al., 2005), probably for prefrontal cortex dysfunction during WM processing (Anticevic et al., 2013b). This is in line with recent evidence showing increased prefrontal activity during WM processing in siblings (Callicott et al., 2003; Seidman et al., 2006; Rasetti et al., 2014) and offspring of patients (Bakshi et al., 2011). However, FP coupling during WM processing seems to be reduced in genetic high risk subjects (Keshavan et al., 2002), although this result must be viewed with caution because the study sample size was quite small. A possible explanation for this inconsistency may be differences in global functioning; it has been shown that only those offspring of psychotic patients with low global functioning (but not those with high global functioning) showed reduced prefrontal activity during WM-related processing compared with healthy controls (Diwadkar et al., 2011).

### Non-help-seeking individuals with psychotic-like experiences

Cognitive deficits already occur in non-help-seeking individuals with psychotic-like experiences. In particular, children with psychotic experiences show defective processing speed during cognitive tasks, which is predictive for their psychotic experiences (Niarchou et al., 2013). However, no explicit WM fMRI study has yet been conducted in non-help-seeking individuals with psychotic-like experiences, but studies using different tasks also showed increased FP activity in non-clinical subjects with auditory hallucinations (Sommer et al., 2008; Diederen et al., 2012). Increased frontal activity during theory of mind tasks have also been observed in individuals with psychotic-like experiences, which suggests that there are compensatory mechanisms to achieve normal behavioral performance (Modinos et al., 2010b; Van Der Meer et al., 2013). Interestingly, the increased FP connectivity during WM processing in individuals with psychotic-like experiences and genetic high-risk subjects is consistent with the increased FP resting state connectivity in individuals with occasional psychosis-like experiences (Orr et al., 2014), but contrasts with the reduced FP connectivity in clinical high-risk subjects. A possible explanation for the direction of WM-induced functional connectivity could be the state-related psychopathological symptoms they experienced (Schmidt and Borgwardt, 2013). Although these non-help-seeking subjects exhibit psychotic-like experiences (Orr et al., 2014), which are negatively related to WM capacity and moderated by age (Ziermans, 2013), they do not force them to seek help. In contrast, clinical high-risk subjects represent help-seeking adolescents, who by definition may already be manifesting established signs of attenuated psychosis (Yung et al., 2004). Although clinical high-risk individuals do not inevitably transit to a full-threshold psychotic illness, they are in need-for-care and with an increased risk of developing a psychotic disorder (Wood et al., 2008). Given that WM deficits become progressively worse over the course of the disorder, with the most severe impairments in established psychosis at the upper end of the continuum (Brewer et al., 2006; Hill et al., 2013), the increase in FP connectivity in individuals with psychotic-like experiences may indeed reflect a compensatory mechanism to counteract these latent (cognitive) impairments. It is therefore important to follow-up these subjects, given that psychotic-like experiences can be indicative of reduced WM capacity in early adulthood, which in turn may reflect an increased risk for psychosis and a greater need for targeted intervention (Ziermans, 2013). Within this framework, the reduced WM-related FP connectivity in clinical high-risk subjects and patients with chronic or first-episode psychosis might reflect already impaired cognition (Friston and Frith, 1995), whereas the increased FP connectivity in subjects with a genetic risk or psychosis-like experiences may reflect a compensatory effect in response to the emerging cognitive deficits. However, this is clearly a speculative observation at the present time and warrants further investigations.

## Summary of functional and structural FP connectivity findings

Numerous studies provide evidence for reduced functional and structural FP connectivity across the psychosis spectrum, suggesting that this pathway is one of the most vulnerable for the emergence of psychosis. It is crucial that functional and structural FP connectivity measures in these regions are often progressively reduced from healthy controls via high risk subjects to patients with first episode psychosis and then with established psychosis. Such a symptom-based dissection of the psychosis continuum has recently been supported by more sophisticated whole-brain network analyses. However, several investigations also reported increased FP connectivity, mainly in genetic high risk subjects and non-help-seeking individuals with psychosis-like experiences. Further studies must investigate whether this increase reflects a compensatory mechanism. Table 2 gives a selective overview of functional and structural FP connectivity findings in each domain in the psychosis continuum.

**Table 2:**
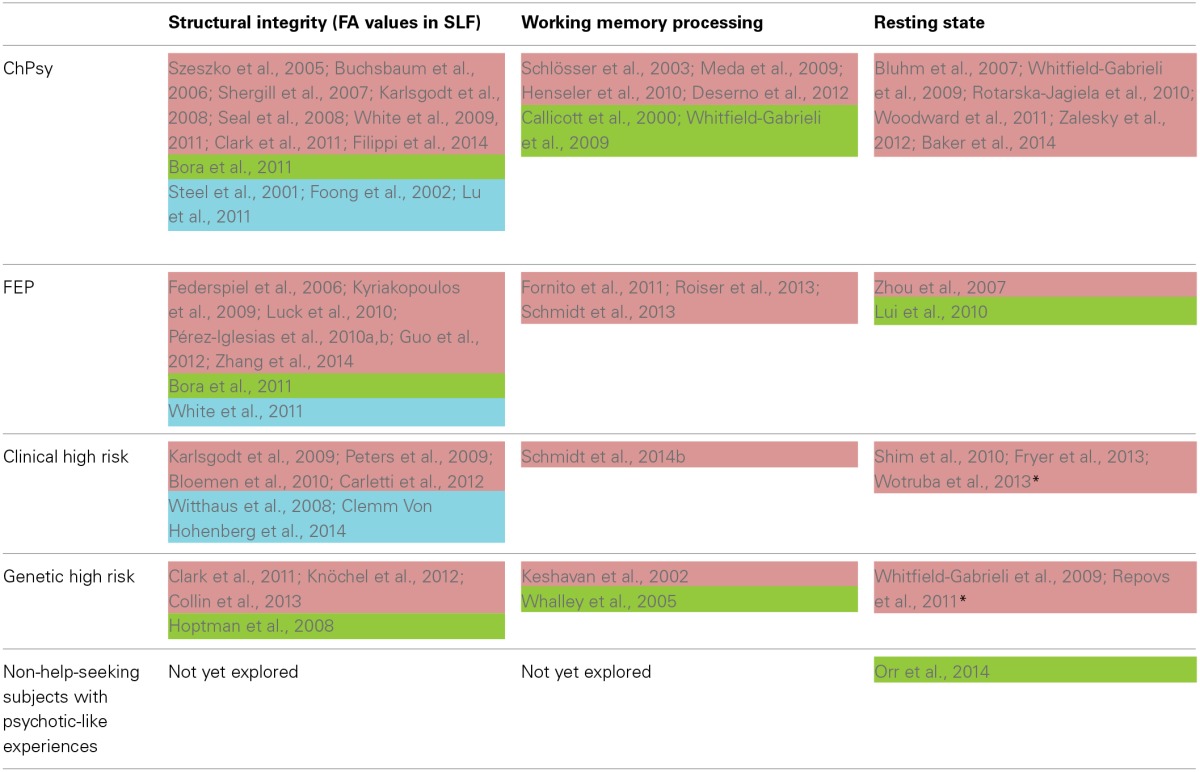
**Selective overview of studies addressing FP connectivity across the psychosis continuum relative to healthy subjects**.

*Red color reflects decreased connectivity, green color indicates increased connectivity and blue means non-significant differences in fronto-parietal connectivity relative to healthy control subjects ChPsy, chronic psychosis; first episode psychosis; SLF, superior longitudinal fasciculus; FA, fractional anisotropy*.

^*^These studies reported reduced inter-network connectivity to the FP network.

## NMDAR function and FP dysconnectivity in psychosis

In this brief chapter, we will discuss a potential pharmacological mechanism underlying aberrant FP connectivity in psychosis. The reductions in WM-induced modulation of FP connectivity have often been interpreted as being a result of the abnormal regulation of N-methyl-D-aspartate receptor (NMDAR)–dependent synaptic plasticity (Deserno et al., 2012; Schmidt et al., 2013). This interpretation is based on theoretical reflections proposing that disordered brain connectivity in psychosis results from abnormal regulation of NMDAR–dependent synaptic plasticity by modulatory transmitters like dopamine, acetylcholine, or serotonin (Stephan et al., 2009a). This regulatory effect of modulatory transmitters on NMDAR-dependent synaptic plasticity (for specific mechanisms underlying this modulation see Stephan et al., 2009a) distinguishes the dysconnection hypothesis from other pathophysiological theories of schizophrenia that postulate impaired NMDAR function alone (Javitt and Zukin, 1991; Olney and Farber, 1995). Numerous genes implicated in schizophrenia also converge on glutamatergic pathways (Allen et al., 2008; Walsh et al., 2008). For example, the glutamate-related genes NRG1 and DTNBP1 are involved in both building long-range connections during development and in regulating synaptic plasticity (Harrison and Weinberger, 2005). This is important, because any impairment in synaptic plasticity would affect the way long range connections are established in the developing brain, given that the strength of functional coupling between two neurons determines whether their connection survives developmental pruning (Hua and Smith, 2004).

With regard to the FP pathway, a recent study suggests that psychotic patients have glutamate-related dysregulation of the brain network supporting cognitive control (Falkenberg et al., 2014). The NMDAR antagonist ketamine induces psychotic-like experiences that are highly reminiscent of those observed in schizophrenia (Krystal et al., 1994, 2005; Schmidt et al., 2012), so it is intriguing that this also reduces diffusivity in the superior longitudinal fasciculus (Edward Roberts et al., 2014). It has further been demonstrated that ketamine reduced the negative correlation between the FP system and the default network in healthy volunteers, while the degree of this disruption predicted task performance (Anticevic et al., 2012). Furthermore, ketamine reduced accuracy on a spatial WM task and connectivity in a prefrontal network bilaterally, and region-specific reductions in connectivity were related to WM performance in healthy subjects (Driesen et al., 2013). These findings underpin the importance of the NMDAR for WM processing (Karlsgodt et al., 2011), in keeping with animal models reporting aberrant WM function after the inhibition of glutamatergic receptors (Timofeeva and Levin, 2011; Arnsten et al., 2012). However, recent data suggest that acute ketamine-induced alterations in brain network connectivity do not parallel those seen in chronic psychosis (Dawson et al., 2014). More evidence is needed to draw valid conclusions about glutamatergic synaptic physiology across different psychosis stages, in particular changes in NMDAR functioning.

## Limitations of current approaches

Despite the considerable effort made in neuroimaging studies to detect abnormal connectivity patterns across different psychosis stages, there is no reliable neurobiological marker of any clinical utility in the psychosis spectrum to date. This can probably be explained on the one hand by significant differences in the analytical approaches across studies and by problems in the interpretation of the results on the other. We briefly present some of these limitations, although it is beyond the scope of this paper to comprehensively address all of them. In graph analysis, the selection of the included brain regions or nodes —the parcellation of the brain —is one of the most critical steps and hampers comparisons among studies. The nodes should represent distinct, functionally homogeneous neural elements or brain regions. However, in the absence of any gold standard for large scale parcellation of the brain, the nodes are typically arbitrarily defined by a variety of methods (Fornito et al., 2012). The nature of links (i.e., connections), whether binary or weighted, also influence connectivity findings (Rubinov and Sporns, 2010). Another general issue is that graph theory provides a bunch of connectivity indexes that can be used in brain networks; it will be important to understand the relations between them and how they are changed in psychosis (Bullmore and Vértes, 2013). Furthermore, structural findings (either analyzed with conventional or graph theory analysis) in patients are difficult to interpret due to the non-specific nature of FA values, i.e., it is unclear whether they reflect neuroinflammation or axonal degeneration (for a comprehensive review on pitfalls in terms of methods and interpretations of DTI findings see O'donnell and Pasternak, 2014). Free-water imaging on DTI data might provide a possible approach to elucidate the exact underlying microstructural basis of white matter changes in psychosis (Pasternak et al., 2012). Many investigations of functional connectomics in psychosis have been performed in the resting state (Alexander-Bloch et al., 2013; Argyelan et al., 2014) and only a few during tasks (Lord et al., 2011). It thus important to disentangle which (dys)connectivity only occurs in a specific psychological context (i.e., task-specific) and which are context-independent and reflect more general impairments (Fornito et al., 2012). In contrast to whole-brain graph analysis, the limitations of DCM analyses lie in the simplified neuronal network model, which often includes only a relatively small number of brain regions. The regions are included on the basis of task-induced activity and effective connectivity findings from DCM analysis and can thus only be compared across studies if exactly the same paradigms have been conducted. In other words, model-based assays can never fully capture a comprehensive neural network that fully considers a specific psychological process. The numbers of included regions are often limited, as model fitting is computationally demanding (Penny et al., 2004). Nevertheless, the restricted model space in DCM analysis can also be an advantage if the included regions comprise highly sensitive clinical data, which are not confounded by unimportant and noisy data from other regions (which is a potential danger of whole-brain graph analysis).

Furthermore, inconclusive findings from patient studies may often reflect the clinical heterogeneity of patient samples, the fact that the patients enrolled were treated with antipsychotic drugs (for different periods) (Fusar-Poli et al., 2013c) and other confounding factors such as comorbidities (Modinos et al., 2014). This is particularly relevant for ultra-high risk samples, as these exhibit high clinical heterogeneity (Fusar-Poli and Van Os, 2013; Simon et al., 2013, 2014; Fusar-Poli et al., 2013b). Furthermore, patient and control samples are often not well matched for intellectual functions and educational level, which might influence imaging results. Cross-sectional designs further hampered inferences about how brain connectivity abnormalities develop over the course of the illness. There are additional problems relating to publication and reporting biases of neuroimaging studies (Fusar-Poli et al., 2014a).

## Future research challenges

**Figure 1:**
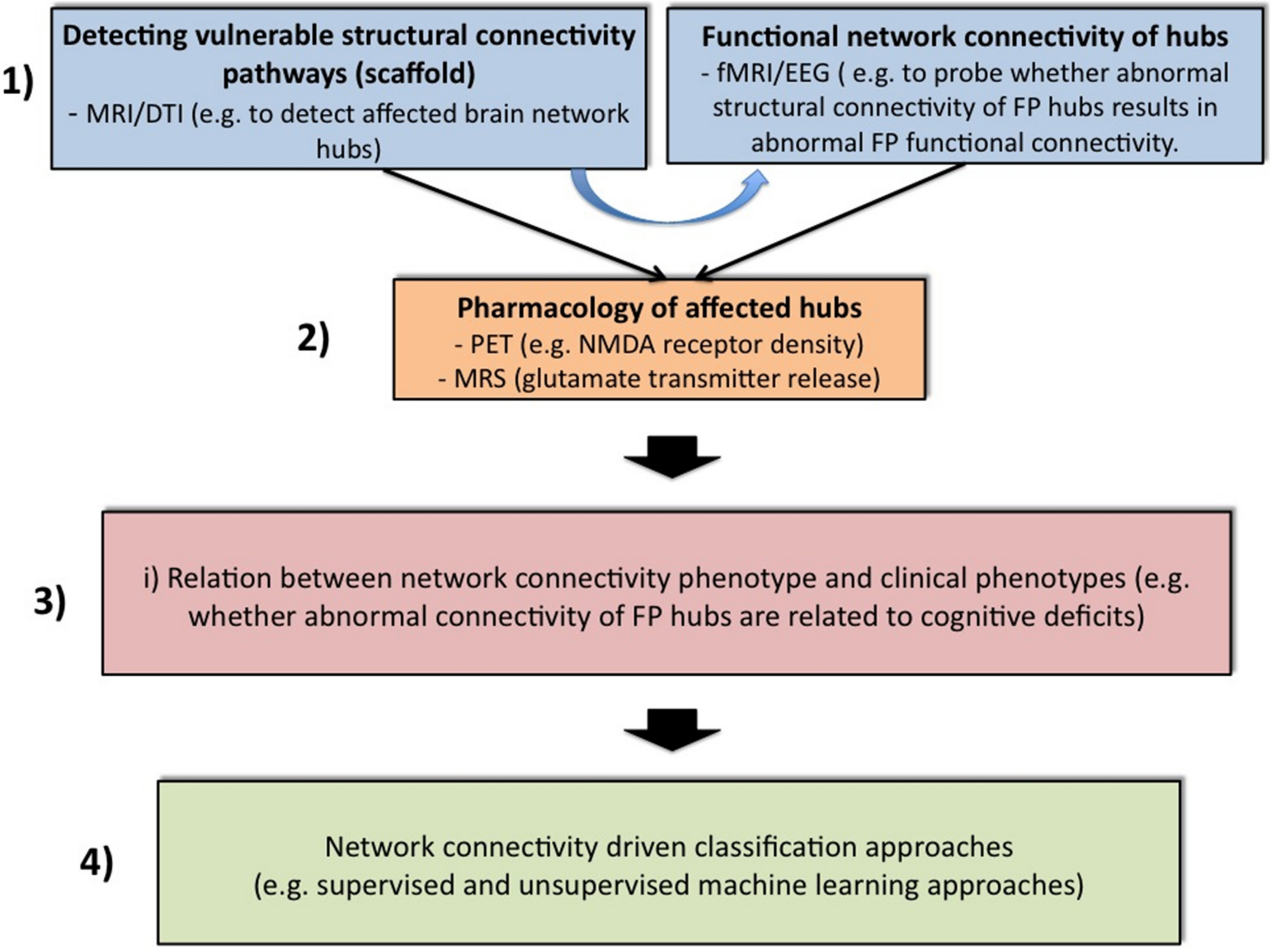
**Four challenging steps toward classification of the psychosis continuum driven by network connectivity**. The purpose of block 1 is to apply structural [magnetic resonance imaging (MRI) and diffusion tensor imaging (DTI)] and functional [functional MRI (fMRI) and electroencephalogram (EEG)] measurements across all stages of the psychosis continuum. Structural network abnormalities should thereby serves as a scaffold to find vulnerable hub connections. It will be attempted to relate these to functional connectivity estimates. In a second step, specific imaging methods such as positron emission tomography (PET) or magnetic resonance spectroscopy (MRS) can be applied to elucidate glutamatergic neurotransmission in these pathways. For instance, PET challenges might be used to explore specific receptor densities at putative vulnerability connections (e.g., the NMDAR profile at frontal hubs), while MRS can be used to assess the release of specific neurotransmitters (e.g., glutamate). After the establishment of a comprehensive network connectivity phenotype, an attempt is made in Block 3 to detect potential candidate sub-networks of high clinical relevance. Thus, this step aims at reducing complexity and increasing selectivity. For example, one conceivable approach would be to restrict the whole brain network to specific hubs that are related to cognitive impairments. This may lead to the formulation of different subtypes of network connectivity mechanisms. These models can be guided for example by the question whether different expressions of connectivity strengths within a specific candidate network contribute to different degrees of cognitive impairments. These models can then be entered into suitable multivariate classifiers with the hope of splitting the psychosis continuum into meaningful subgroups (block 3).

Further analytical steps are also needed to tackle these limitations and to improve the biological- and model-based diagnosis of psychiatric spectrum diseases (Cuthbert and Insel, 2010; Stephan and Mathys, 2014), with the incorporation of the findings on FP connectivity (cf. Table 2). Figure 1 gives a speculative graphical overview of these critical key steps to achieving a network connectivity-driven classification of the psychosis continuum.

In the first and very important step, we have to elucidate whether functional dysconnectivity in psychosis has a structural basis and how such a putative structure-function relation characterizes each domain of the psychosis continuum, i.e., chronic psychosis, early psychosis, clinical high risk, genetic high risk and general population with psychotic experiences. For this structural-functional approach, multiple imaging modalities are required to explore not only connectivity properties within a specific network such as the FP system, but also to assess comprehensive connectivity maps across the whole brain. For model-based investigations of effective connectivity, i.e., DCM analysis, the definition of the model space should not only be motivated by task-induced brain activity and between-group differences in these activities, but also by evidence for abnormalities in structural connectivity in specific regions. This is important, as it is likely that functional connectivity over long time periods of 5 to 10 min reflects underlying structural connectivity (Park and Friston, 2013). Graph theoretical network analysis also offers promising perspectives for this purpose (Bullmore and Sporns, 2009; Filippi et al., 2013). For example, a recent network study found increased coupling between structural whole-brain connectivity and resting state functional connectivity in patients with psychosis. The increased correlation may suggest that the illness leads to functional interactions that are more directly related to the underlying anatomical connectivity of the brain and may be indicative of more stringent and less dynamic brain function in patients (Van Den Heuvel et al., 2013). Another study found that psychotic patients showed decreased functional connectivity and impaired white matter integrity in a distributed network encompassing frontal, temporal, thalamic, and striatal regions (Cocchi et al., 2014). Along these lines, recent work summarizing results from structural correlation studies, diffusion-imaging tractography studies, and functional correlation studies found strong evidence for network abnormalities of prefrontal hubs, and moderate evidence for network abnormalities of limbic, temporal, and parietal hubs (Rubinov and Bullmore, 2013b).

In a second step after the detection of connectivity pathways that are vulnerable with respect to structure and function, specific imaging methods can be applied to elucidate glutamatergic transmission in these pathways. For instance, positron emission tomography challenges might be used to explore specific receptor densities at putative vulnerability connections (e.g., the NMDAR profile at frontal hubs). Within this perspective, it has been shown that functional connectivity from the prefrontal cortex to thalamus and nucleus accumbens correlated positively with prefrontal glutamate concentrations. The correlations involving prefrontal glutamate and prefrontal-related functional connectivity were mirrored by correlations with structural connectivity (Duncan et al., 2013). Linking such experimental investigations with computational models may help to bring together evidence from the receptor to the system levels in psychosis (Adams et al., 2013; Anticevic et al., 2013a).

In a third step, it should be studied how these comprehensive network connectivity phenotypes are associated with behavioral, cognitive and genetic phenotypes across each domain of the psychosis continuum. This may allow the detection of potential candidate network circuits with particularly high clinical sensitivity. Such studies have already been performed, for example on the relation between abnormal brain signals, cognition and chemistry in early stages of psychosis (Schmidt and Borgwardt, 2014). Having in mind that the psychosis continuum describes a trajectory with increasingly severe symptoms, including cognitive impairments (Yung et al., 2012; Fusar-Poli et al., 2013a), a robust relation between aberrant network organization and cognitive functioning might facilitate the dissection of different illness stages. For example, altered network organization among frontal and parietal brain areas in schizophrenia patients has been related to WM performance (Bassett et al., 2009), whereas global connectivity in the lateral prefrontal cortex, involving connections both within and outside the FP network, predicts cognitive control and intelligence (Cole et al., 2012). Variation at one RGS4 single nucleotide polymorphism that has been previously associated with psychosis (rs951436) impacts fronto-parietal and fronto-temporal network coupling during WM and results in regionally specific reductions in the structural volume of gray and white matter in individuals carrying the A allele (Buckholtz et al., 2007). Another psychosis-related gene, the ZNF804A rs1344706, is also associated with prefrontal brain connectivity (Mothersill et al., 2012).

In a final determining step, we propose exploration of whether the pattern of network connectivity differs between domains in the psychosis continuum. One possible approach for disease classification and prediction would be to use supervised learning algorithms such as support vector machine (SVM), which have already been applied in early psychosis detection (Koutsouleris et al., 2012, 2014; Borgwardt et al., 2013). However, applying multivariate methods with whole-brain connectomics data requires some way of reducing dimensionality, as the connectomes are massively large. This problem has been addressed in a recent study, that employs a multivariate approach based on the SVM and regularization methods that exploit the 6-D spatial structure of the functional connectome (Watanabe et al., 2014). In this regard, a very recent study demonstrated that structural connectomes from DTI data in combination with functional connectomes from resting state fMRI data provided classification accuracy of 100% between healthy controls and chronic psychotic patients (Zhu et al., 2014). Another conceivable procedure, which actually only requires an additional step before running classification algorithms, is to use all these neurophysiological features (structure, function and chemistry) and formulate biophysiologically informed generative models (e.g., DCMs) (Stephan and Mathys, 2014), which, with formal selection procedures such as Bayesian model selection (Stephan et al., 2009b), might lead to dissection of the psychiatric spectrum diseases. For example, one could build different FP network models in which cognitive load modulates connectivity strength between the regions in the model at different sites. These models could then be fed into a multivariate classification algorithm. At best, the unsupervised classifier may find some connectivity clusters, which separate subjects into clinically meaningful subgroups. A very recent study provides a concrete example for such an approach by demonstrating how psychotic patients can be dissected into three subgroups defined in terms of neurophysiological mechanisms specified by a generative model of network dynamics (DCMs). It is impressive that the three neurophysiologically defined subgroups mapped onto three clinically distinct subgroups, distinguished by significant differences in negative symptom severity (Brodersen et al., 2013). In other words, instead of simply entering all possible neurophysiological features into the classification algorithm, all this information should be included in a generative model, such as DCM. Given that functional connectivity is highly constrained by structural connectivity (Park and Friston, 2013) and that structural connectivity can be influenced via NMDAR-dependent synaptic plasticity (Stephan et al., 2006), one should include only brain regions in the generative model from which such alterations have been reported in psychosis patients. In such an approach, all neurophysiological parameters are embedded in one model. By applying multivariate classifiers to measures of gray matter volumes, more than 80% accuracy has been achieved in distinguishing clinical high-risk subjects with later transition to psychosis from those without transition (Koutsouleris et al., 2012). Given that the accuracy of distinction between healthy controls and psychotic patients increases when using DCM-based effective connectivity estimates (78%), rather than functional connectivity (62%) or local activity estimates within these regions (55%) (Brodersen et al., 2013), feeding formally described mechanisms of network connectivity phenotypes into classification approaches should be more accurate than using simple isolated features. Replication and large-scale longitudinal studies are then required to validate diagnostic reliability and treatment responses.

## Conclusion

Brain imaging may provide a powerful tool to improve the specificity and validity of an early diagnosis and to sustain preventive intervention prior to the onset of illness (Fusar-Poli et al., 2012b). Although no reliable neuroimaging marker of any clinical utility in the psychosis spectrum has yet been established, the assessment of network connectivity information from fMRI and DTI data might bring us one step closer to this goal. The present paper tried to demonstrate this potential using the example of FP network connectivity. One promising approach for the detection of network connectivity endophenotypes is the computation of whole-brain connectivity patterns using graph theory. This technology allows the study of network properties from a global perspective that may help to detect potential network endophenotypes for psychosis spectrum disorders (data-driven approach) (Van Den Heuvel and Fornito, 2014). The organization of network hubs seems to be especially important, given that recent studies have suggested that abnormal connectivity of brain hubs may be a core aspect of the disorder (Rubinov and Bullmore, 2013a,b; Van Den Heuvel and Sporns, 2013). An alternative strategy is to search endophenotypes in terms of biophysically constrained models of effective connectivity (e.g., DCM). This procedure is particularly useful in testing synaptic mechanisms that might underlie aberrant patterns of correlations (hypothesis-driven approach). This is important, as pharmacological treatments exert their effect at the synaptic level. It is important that these two concepts should be applied in a complementary fashion (Park and Friston, 2013). For example, graph theoretical analysis of DTI data can be used to detect affected brain circuitries in terms of abnormal structural connectivity properties, which at best also show a relation to clinical endophenotypes. These clinically relevant findings can then be used to formulate the model architecture of generative network models, i.e., the model of effective connectivity is constrained by structural connectivity evidence from graph theory. This improves the biological realism of the model and facilitates a mechanistic understanding of the signals that may increase the accuracy of machine learning engines, as recently suggested (Varoquaux and Thirion, 2014). It is quite clear that extensive work is needed to test the predictive and diagnostic validity of these network techniques, including longitudinal study design and large and well-matched study samples.

### Conflict of interest statement

The Review Editor Dr. Kroken declares that, despite having collaborated with the author Paolo Fusar-Poli, the review process was handled objectively and no conflict of interest exists. The authors declare that the research was conducted in the absence of any commercial or financial relationships that could be construed as a potential conflict of interest.
